# Supplementary material for: Asynchronous magnetic resonance elastography: Shear wave speed reconstruction using noise correlation of incoherent waves
Source: Magn Reson Med. 2022 Oct 27;89(3):990–1001. doi: 10.1002/mrm.29502 (PMC9792433; doi:10.1002/mrm.29502)
Supplement: Supplementary file 1 — DATA S1 MATLAB code used to generate the numerical simulations of Figure 1 [file MRM-89-990-s002.zip › k-Wave/helpfiles/getOptimalPMLSize.html]

getOptimalPMLSize :: Functions (k-Wave)


# getOptimalPMLSize

Find PML size to give the smallest prime factors.

## Syntax

```
pml_sz = getOptimalPMLSize(grid_sz)
pml_sz = getOptimalPMLSize(grid_sz, pml_range)
pml_sz = getOptimalPMLSize(grid_sz, [], axisymmetric)
pml_sz = getOptimalPMLSize(grid_sz, pml_range, axisymmetric)
```

## Description

`getOptimalPMLSize` finds the size of the perfectly matched layer (PML) that gives an overall grid size with the smallest prime factors when using the first-order simulation functions in k-Wave with the optional input `'PMLInside', false`. Choosing grid sizes with small prime factors can have a significant impact on the computational speed, as the code computes spatial gradients using the fast Fourier transform (FFT).

## Inputs

|  |  |
| --- | --- |
| `grid_size` | Grid size defined as a one (1D), two (2D), or three (3D) element vector. Alternatively, can be an object of the `kWaveGrid` class defining the Cartesian and k-space grid fields. |

## Optional Inputs

|  |  |
| --- | --- |
| `pml_range` | Two element vector specifying the minimum and maximum PML size (default = `[10, 40]`). |
| `axisymmetric` | If using the axisymmetric code, string specifying the radial symmetry. Allowable inputs are `'WSWA'` and `'WSWS'` (default = []). This is important as the axisymmetric code only applies to the PML to the outside edge in the radial dimension. |

## Outputs

|  |  |
| --- | --- |
| `pml_opt` | PML size that gives the overall grid with the smallest prime factors. |

## See Also

`fft`
